# Underwater forceps-assisted polypectomy of an appendiceal orifice lesion using a dual-channel gastroscope

**DOI:** 10.1016/j.vgie.2024.09.015

**Published:** 2024-10-04

**Authors:** Mikio Kobayashi, Hideyuki Chiba, Akimichi Hayashi, Yu Ebisawa, Jun Arimoto, Hiroki Kuwabara, Michiko Nakaoka

**Affiliations:** Department of Gastroenterology, Omori Red Cross Hospital, Tokyo, Japan

Endoscopic treatment of lesions located within the appendiceal orifice (AO) has not yet been established; it is difficult to obtain a stable endoscopic field using cold polypectomy and EMR. Underwater EMR sometimes provides a better endoscopic field, but in the narrow space within the AO, snaring can be difficult while keeping the margins in place. As a result, some cases have been reported in which underwater EMR resulted in piecemeal resection.^1^ Herein, we describe a novel treatment for a lesion involving the AO using a combination of forceps-assisted polypectomy and underwater technique, using a dual-channel endoscope, GIF-2TQ260M (Olympus Medical Systems, Tokyo, Japan) (Video 1, available online at www.videogie.org).

A 76-year-old man was admitted to our hospital for polypectomy. An 8-mm lesion was found within the AO (Fig. 1). Narrow-band imaging with magnification showed Japan Narrow-band Imaging Expert Team classification type 1, and sessile serrated lesion was suspected. First, a 10-mm snare (Captivator II, Boston Scientific, Natick, Mass, USA) was inserted through one channel, and a grasping forceps, FG-47L-1 (Olympus Medical Systems) was inserted through the other channel and passed through the snare. Then, under water immersion, the lesion was easier to grasp with the forceps as the result of the buoyancy effect (Fig. 2). The polyp was pulled up with the grasping forceps to ensure that the end of the lesion was secured and snared, and a strip biopsy was performed (Fig. 3). The patient underwent successful en bloc resection (Figs. 4 and 5). No serious adverse events, including appendicitis, were observed after this treatment. Findings of the pathologic examination confirmed a sessile serrated lesion with negative margins (Fig. 6). Three months later, follow-up colonoscopy showed no recurrence.

**Figure 1:**
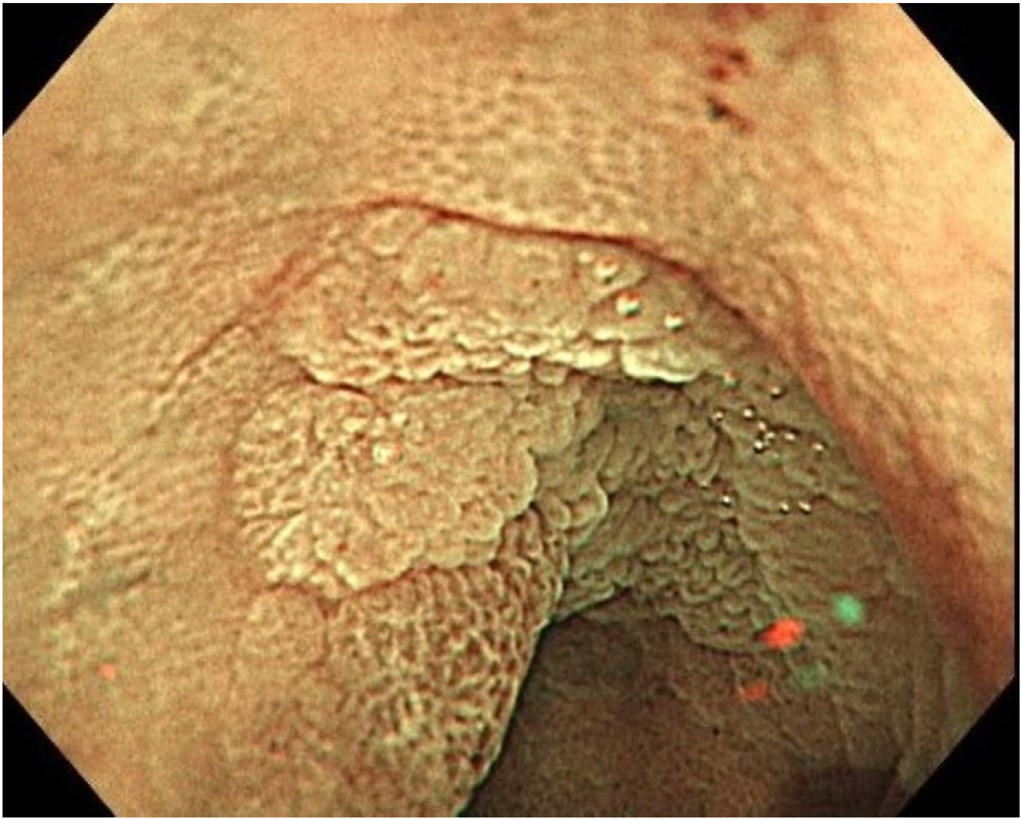
The distal border of the lesion was not visible because the appendicular orifice was collapsed. However, the entire lesion could be observed as the result of the buoyancy effect under water immersion.

**Figure 2:**
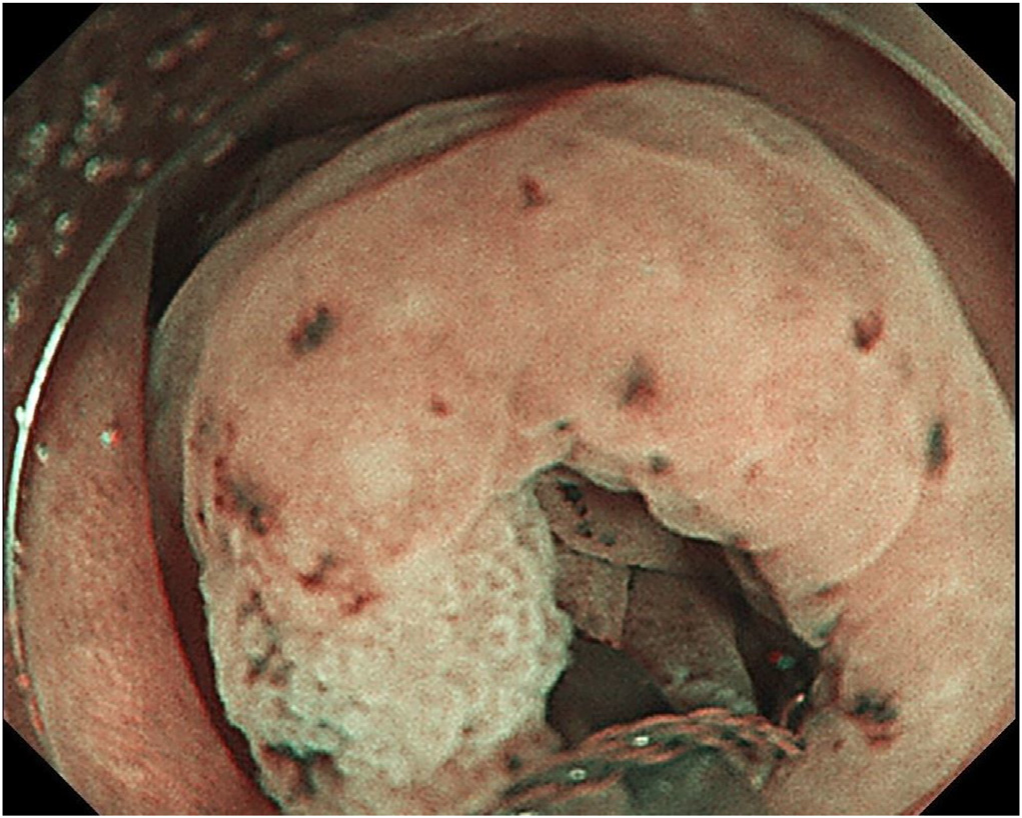
Gripping the lesion to confirm its distal border before snaring.

**Figure 3:**
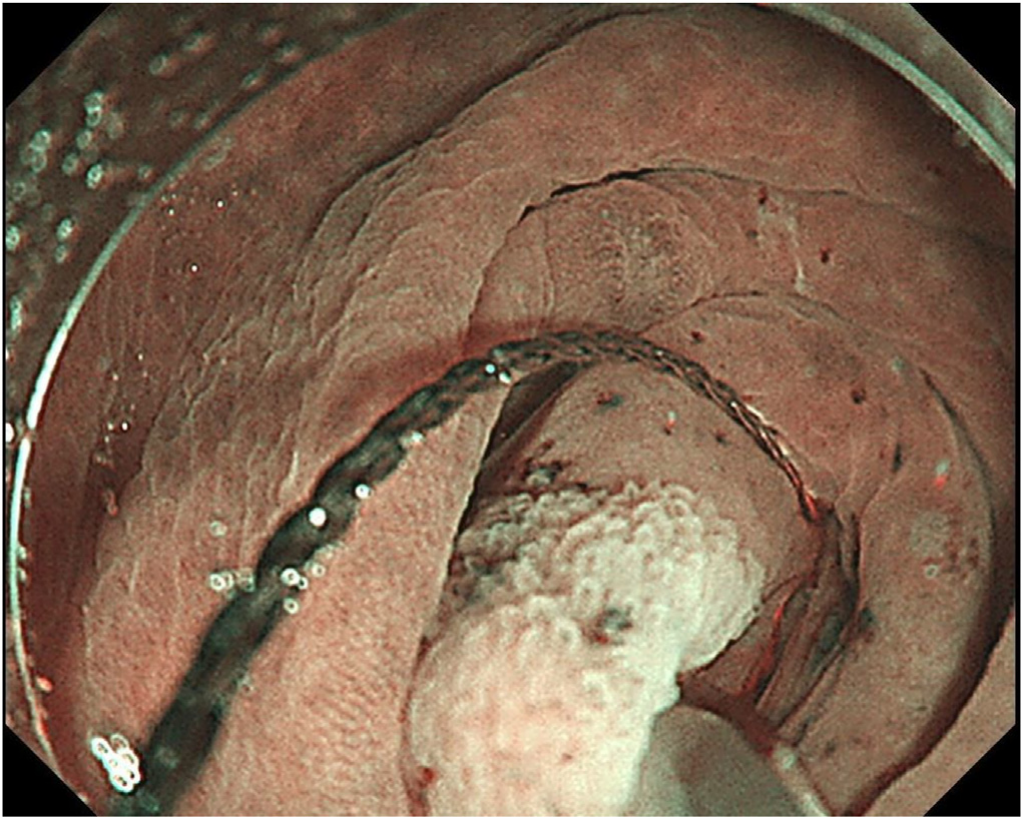
Snaring the lesion while gripping it with forceps.

**Figure 4:**
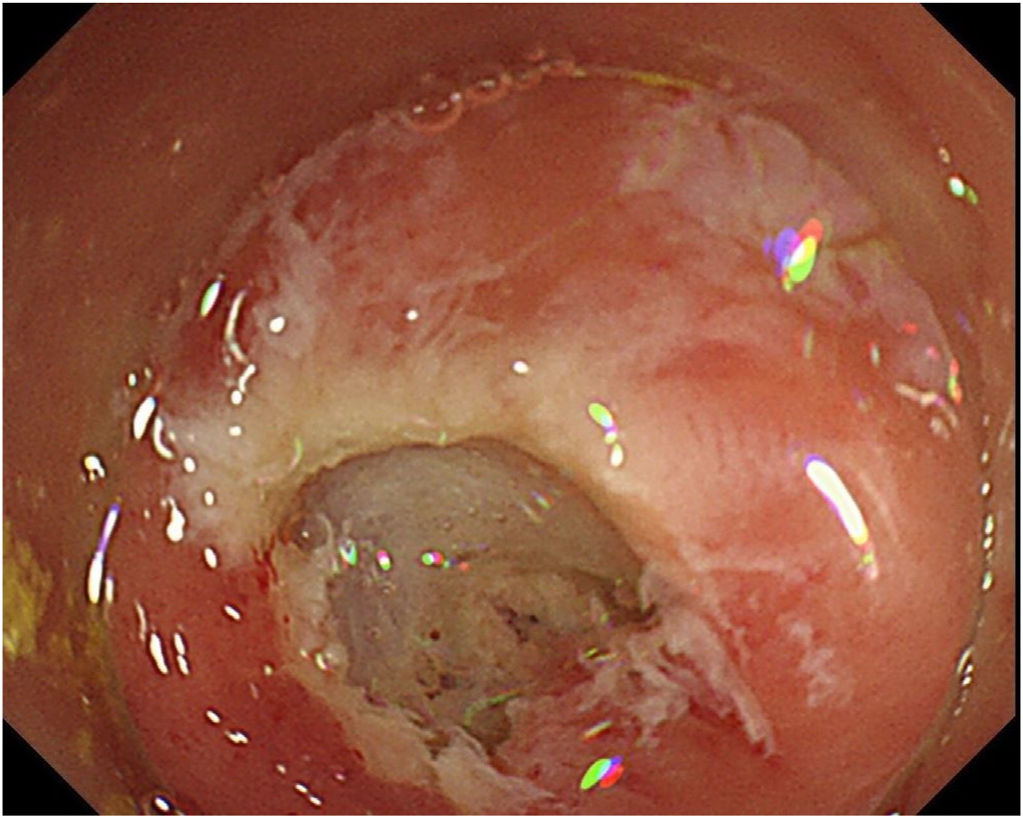
The ulcer floor after underwater forceps-assisted polypectomy.

**Figure 5:**
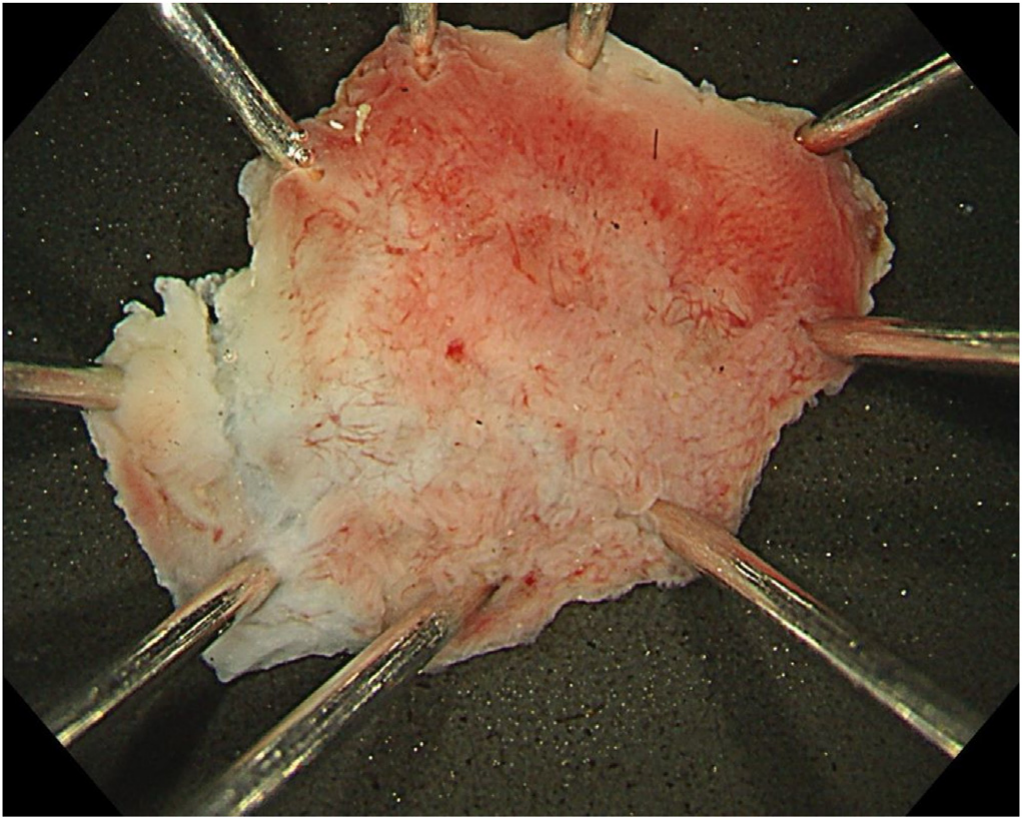
The resected specimen.

**Figure 6:**
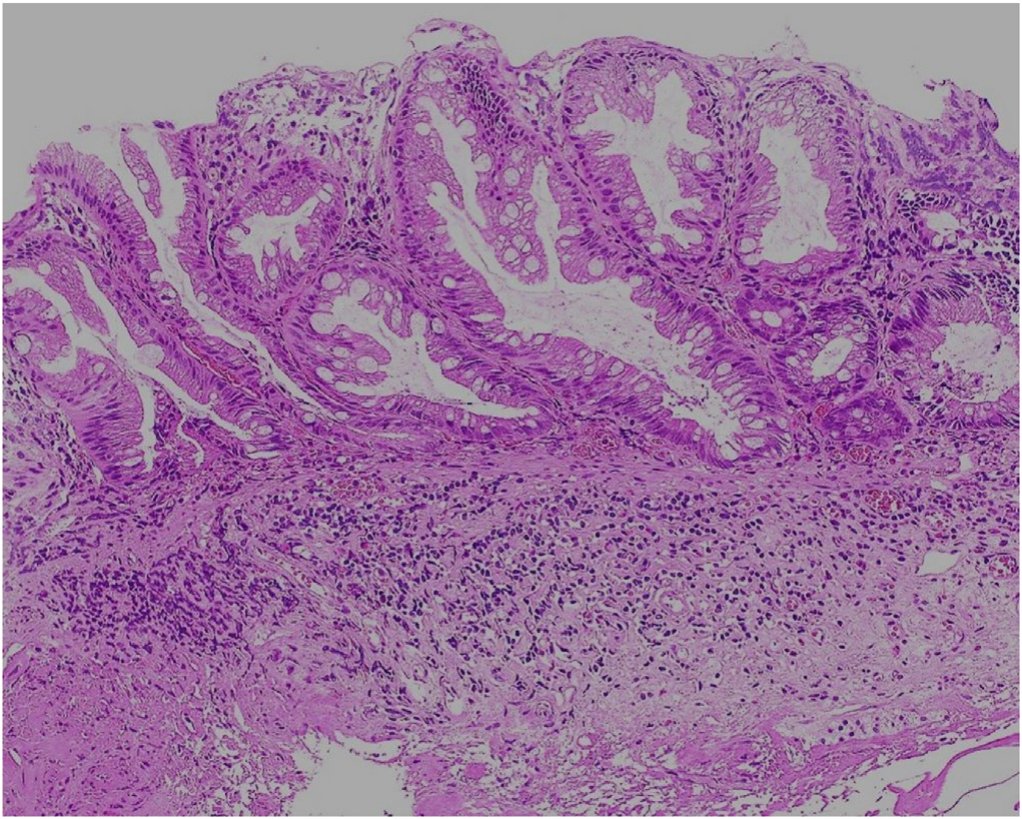
Findings of the pathologic examination revealed a boot-shaped crypt; the lesion was diagnosed as a sessile serrated lesion (H&E, orig. mag. ×100).

In underwater forceps-assisted polypectomy, even when the lesion is depressed at the AO and the distal border is not visible, performing the procedure underwater can improve visibility as a result of the buoyancy effect. Moreover, the distal end can be set and snared accurately while the lesion is grasped to stabilize the endoscopic field. This technique could be one effective option for lesions involving the AO.

## Patient consent

The patient in this article has given written informed consent to publication of the case details.

## Disclosure

All authors disclosed no financial relationships.

## Supplementary data

Video 1 Underwater forceps-assisted polypectomy of an appendiceal orifice lesion, using a dual-channel endoscope.
